# Novel digital droplet inverse PCR assay shows that natural clearance of hepatitis B infection is associated with fewer viral integrations

**DOI:** 10.1080/22221751.2025.2450025

**Published:** 2025-01-03

**Authors:** Dong Li, Vikki Ho, Chiao-Fang Teng, Hung-Wen Tsai, Yuanyuan Liu, Sarah Bae, Harout Ajoyan, Jochen M. Wettengel, Ulrike Protzer, Brian S. Gloss, Rebecca J. Rockett, Rafid Al Asady, Jane Li, Simon So, Jacob George, Mark W. Douglas, Thomas Tu

**Affiliations:** a Storr Liver Centre, The Westmead Institute for Medical Research, Westmead Hospital and The University of Sydney, Westmead, NSW, Australia; b Graduate Institute of Biomedical Sciences, China Medical University, Taichung, Taiwan; c Program for Cancer Biology and Drug Discovery, China Medical University, Taichung, Taiwan; d Organ Transplantation Center, China Medical University Hospital, Taichung, Taiwan; e Department of Pathology, National Cheng Kung University Hospital, Tainan, Taiwan; f Institute of Virology, Technische Universität München/Helmholtz Zentrum München, Munich, Germany; g German Center for Infection Research (DZIF), Munich Partner Site, Munich, Germany; h Scientific Platforms, The Westmead Institute for Medical Research, The University of Sydney, Westmead, NSW, Australia; i Centre for Infectious Diseases and Microbiology–Public Health, Westmead Hospital, Westmead, NSW, Australia; j Department of Radiology, Westmead Hospital, Westmead, NSW, Australia

**Keywords:** HBV DNA integration, functional cure, fine needle aspiration, digital droplet PCR, HBsAg

## Abstract

Hepatitis B virus (HBV) DNA integration into the host cell genome is reportedly a major cause of liver cancer, and a source of hepatitis B surface antigen (HBsAg). High HBsAg levels can alter immune responses which therefore contributes to the progression of HBV-related disease. However, to what extent integration leads to the persistent circulating HBsAg is unclear. Here, we aimed to determine if the extent of HBV DNA integration is associated with the persistence of circulating HBsAg in people exposed to HBV. We established a digital droplet quantitative inverse PCR (dd-qinvPCR) method to quantify integrated HBV DNA in patients who had been exposed to HBV (anti-HBc positive and HBeAg-negative). Total DNA extracts from both liver resections (n = 32; 14 HBsAg-negative and 18 HBsAg-positive) and fine-needle aspirates (FNA, n = 10; 2 HBsAg-negative and 8 HBsAg-positive) were analysed. Using defined *in vitro* samples for assay establishment, we showed that dd-qinvPCR could detect integrations within an input of <80 cells. The frequency of integrated HBV DNA in those who had undergone HBsAg loss (n = 14, mean ± SD of 1.514 × 10^−3 ^± 1.839 × 10^−3^ integrations per cell) was on average 9-fold lower than those with active HBV infection (n = 18, 1.16 × 10^−2 ^± 1.76 × 10^−2^ integrations per cell; *p* = 0.0179). In conclusion, we have developed and validated a highly precise, sensitive and quantitative PCR-based method for the quantification of HBV integrations in clinical samples. Natural clearance of HBV is associated with fewer viral integrations. Future studies are needed to determine if dynamics of integrated HBV DNA can inform the development of curative therapies.

## Introduction

Chronic infection with the Hepatitis B virus is the single most common driver of liver cancer. Worldwide, over 360 million people are chronically infected with HBV, which causes ∼1.1 million deaths each year due to liver cancer or liver failure [1]. The current treatments (reverse transcriptase inhibitors) are taken for indefinite periods as they simply suppress virus replication, but do not cure the infection [2]. The risks of life-threatening disease and the lifelong persistence of HBV infection lead to considerable stigma for people living with chronic hepatitis B, causing impacts to quality of life [3]. Thus, one of the main goals of HBV research is developing a finite-term treatment strategy that eliminates both liver disease progression and viral persistence.

In a consensus statement from the HBV research community, the loss of serum HBV surface antigen (HBsAg) has been selected as one of the primary endpoints for novel therapies (defining as functional cure) [4]. The main rationale for this state is drawn from the natural history of chronic HBV infection (i.e. HBsAg loss), where a dramatic reduction in liver disease progression and hepatocellular carcinoma (HCC) risk accompanies the loss of serum HBsAg [5,6].

This loss of serum HBsAg reflects both an increase in the integration rate of neutralizing antibodies against HBsAg as well as a reduction of HBsAg-secreting cells in the liver. However, despite the loss of detectable HBsAg in the blood, HBV forms that encode HBsAg persist in the liver. First, covalently closed circular DNA (cccDNA, the episomal virus template for all HBV products) has been detected in both animal models of acute HBV infection (in woodchucks and chimpanzees) [7–9], as well as in patients who have spontaneously cleared chronic HBV infection [10–13]. Some of these cccDNA molecules are “defective” and contain mutations rendering them replication incompetent [14]. However, a subset of replication-competent cccDNA forms are still present and can re-initiate HBV infection in cases of immunosuppression [15].

In addition, integrated HBV DNA can be detected in animal models and in humans after HBsAg loss. These forms of HBV DNA inserted into the host cell genome are replication-defective but can maintain HBsAg expression [16,17] (indeed becoming the main source of HBsAg in latter stages of disease [17–20]). At initial infection, integrations occur in ∼1 in 10^4^–10^5^ cells [21–23], but can become at least 10-fold more frequent through the hepatocyte clonal expansion that occurs during immune-mediated cell death and subsequent liver turnover [24–27].

Given this, an immune response directed against both cccDNA and integrated forms of HBV DNA may be necessary to induce HBsAg loss [28–30]. While levels of cccDNA have been quantified in HBsAg-negative patients [31], the frequency of integrated HBV DNA has not been well characterized due to technical difficulties in quantifying this form.

Various approaches have been used to characterize HBV integration sites in liver tissues, including Southern blot hybridization [32–34], *Alu*-PCR [35–38] and recent high-throughput sequencing technology such as whole genome sequencing [39–42] and RNA sequencing [43,44]. These methods have unique capabilities but do not have the sensitivity to detect single copies of integrations [16]. Inverse nested PCR (invPCR) is a specialized technique that allows the amplification of unknown human DNA regions adjacent to integrated HBV sequence [22,27,45]. invPCR involves a first step of DNA digestion using specific restriction enzymes, followed by DNA circularization of cleavage products through self-ligation, then amplification using specific primers that can amplify the cellular DNA sequences proximal to the HBV integration [46,47], Our team has demonstrated invPCR has high sensitivity, allowing absolute quantification of integrations down to single copy virus-cell junctions [21,48]. However, quantification with invPCR relies on manual serial dilution and lacks precision. We therefore built on the existing invPCR technique and established a new streamlined and highly quantitative assay based on digital droplet PCR (ddPCR) technology to simplify the study of HBV DNA integration.

Using this digital droplet quantitative inverse PCR (dd-qinvPCR) method, we compared HBV DNA integration rates in people with chronic HBV infection with those who have cleared HBsAg (functional cure) to determine the contribution of integrations to the persistence of circulating HBsAg. As our key result, we found that the natural clearance of HBV (i.e. HBsAg loss after a confirmed chronic HBV infection without any external therapy) is associated with fewer viral integrations.

## Methods

### Patient liver tissues

Resection liver tissue for this study was sourced from 32 Taiwanese patients previously exposed to HBV who underwent liver surgery (patient details in Table 1). All tissue donors gave written informed consent for the use of liver specimens excess to diagnostic needs, and the protocol was approved by the ethics review committee of the Institutional Review Board (IRB) of National Cheng Kung University Hospital (IRB protocol number: A-ER-105-437). All patients were confirmed as: (1) anti-HBc positive, indicating prior exposure to HBV; and/or (2) HBsAg-positive, indicating current HBV infection. They were split into two groups: Group 1 – HBsAg-positive chronic HBV patients (n = 18); and Group 2 – HBsAg-negative patients (n = 14). For patients in Group 2, HBsAg-negativity had been observed for at least 2 years and all were confirmed to have undetectable plasma HBV DNA. The liver tissue used was collected excess to clinical need after resection for either liver cancer or metastatic colorectal cancers in the liver. All samples analysed were from non-tumour liver collected at surgery, snap-frozen and stored at −80°C prior to analysis.

**Table 1.** Profile of clinical participants.

Sample Type	Case	Sex	Age	Cancer	Fibrosis	HBV DNA (IU/mL)	AVT	AVT duration	HBeAg	HBsAg	anti-HBc	anti-HBe	anti-HBs
Liver resection	1	M	87	HCC	F1	N/A	No	N/A	ND	−	+	ND	ND
	2	F	58	HCC	F1	Not detected	No	N/A	ND	−	+	ND	ND
	3	M	62	HCC	F2	N/A	No	N/A	ND	−	+	ND	+
	4	F	59	HCC	F4	Not detected	No	N/A	−	−	+	+	+
	5	F	70	HCC	F4	N/A	No	N/A	−	−	+	−	+
	6	M	56	HCC	F2	N/A	No	N/A	ND	−	+	ND	ND
	7	M	70	HCC	F1	N/A	No	N/A	ND	−	+	ND	+
	8	M	68	HCC	F2	N/A	No	N/A	ND	−	+	ND	+
	9	M	59	HCC	F2	<20	No	N/A	−	+	−	+	ND
	10	M	45	HCC	F4	5,000,000	No	N/A	−	+	+	−	−
	11	F	56	HCC	F1	63,5000	Yes & stopped	3 years	−	+	+	+	−
	12	M	56	HCC	F4	<20	Yes	7 months	−	+	+	+	−
	13	F	61	HCC	F4	<20	Yes	4 years	−	+	ND	+	−
	14	M	41	HCC	F3	313	No	N/A	−	+	ND	+	−
	15	M	51	HCC	F1	160,000	No	N/A	−	+	ND	+	−
	16	M	48	HCC	F4	8080	No	N/A	−	+	ND	+	−
	17	M	43	HCC	F3	N/A	No	N/A	−	+	ND	+	−
	18	M	69	HCC	F4	<20	Yes	1 year	−	+	ND	+	−
	19	M	64	HCC	F4	171,000	No	N/A	−	+	ND	ND	ND
	20	M	59	HCC	F2	60,500	No	N/A	−	+	ND	ND	−
	21	M	50	HCC	F4	6620	No	N/A	−	+	ND	+	−
	22	F	86	HCC	F3	65.7	No	N/A	−	+	ND	+	−
	23	M	51	HCC	F4	52	Yes	2 weeks	−	+	ND	+	−
	24	F	50	MCRC	F2	N/A	No	N/A	ND	−	+	ND	+
	25	M	35	MCRC	F1	Not detected	No	N/A	ND	−	+	ND	+
	26	M	74	Adeno-carcinoma	F1	N/A	No	N/A	ND	−	+	ND	ND
	27	M	70	MCRC	F3	N/A	No	N/A	ND	−	+	ND	ND
	28	F	69	MCRC	F2	N/A	No	N/A	ND	−	+	ND	+
	29	M	76	MCRC	F1	N/A	No	N/A	ND	−	+	ND	+
	30	M	58	MCRC	F1	94.5	Yes	3 months	ND	+	+	+	−
	31	M	57	MCRC	F1	<20	Yes	5 years	−	+	ND	+	−
	32	F	59	MCRC	F2	39.3	No	N/A	−	+	ND	ND	−
Fine needle aspiration	1	M	73	No cancer	F0	Not detected	Yes	2 years	−	+	ND	+	−
	2	M	43		F0	Not detected	Yes	5 years	−	+	ND	+	−
	3	M	47		F0	22	Yes	2 years	−	+	ND	+	ND
	4	F	57		F0	2340	No	N/A	+	+	ND	−	−
	5	F	55		F0	<10	Yes	3 years	−	+	ND	+	ND
	6	F	46		F0	1,440,000	No	N/A	+	+	ND	−	ND
	7	M	35		F0	4060	No	N/A	−	+	ND	+	ND
	8	M	73		F1	Not detected	No	N/A	−	−	ND	+	+
	9	M	62		ND	Not detected	Yes	> 1 year	−	−	ND	−	−
	10	F	48		F2	13,600	No	N/A	−	+	ND	+	−

Liver fibrosis was quantified by histology for resection tissue and fibroscan for FNAs, and is provided as Metavir Fibrosis score [49].

M: male; F: Female; AVT: antiviral treatment; HCC: hepatocellular carcinoma; MCRC: metastatic colorectal adenocarcinoma.

HBeAg: Hepatitis B e Antigen; HBsAg: Hepatitis B Surface Antigen; anti-HBc: anti-Hepatitis B Core Antibody; anti-HBe: anti-Hepatitis B e Antibody; anti-HBs: anti-Hepatitis B Surface Antibody; HCV Ab: Hepatitis C Virus Antibody.

“+”: Positive; “−”: Negative; ND: not done.

Fine needle aspiration (FNA) samples were collected at Westmead Hospital, Australia from people previously exposed to HBV (clinical details in Table 1). All patients gave written informed consent for the use of liver tissue for research, and the protocol was approved by Western Sydney Local Health District Human Research Ethics Committee (2019/ETH01913). FNA was performed under local anaesthesia and ultrasound guidance using aseptic technique. The skin under the ribs on the right side was infiltrated with local anaesthetic, and then a 22-gauge needle attached to a 5 mL syringe was advanced into the liver. The needle was fanned in the liver several times while maintaining gentle negative pressure on the syringe to aspirate liver cells. The aspirate was transferred into ice cold Dulbecco’s Modified Eagle’s Medium (DMEM), centrifuged at 50 × g, washed twice with 1 mL DMEM, and then stored at −80°C until analysis.

All clinical data were collected and deidentified to protect patient privacy.

### Generation of clones containing integrations

A novel *in vitro* model was used to generate cell lines with *de novo* HBV DNA integrations. HepG2-NTCP (human hepatoma cell line that expresses the NTCP receptor, a gift from Professor Stephan Urban) were maintained in DMEM and infected as per previous protocols [50]. Briefly, HepG2-NTCP cells (seeded at 5 × 10^5^ cells/mL) were infected in 24-well plates, with a replication-deficient reporter HBV encoding for zeocin-resistance (HBV-Zeo), replacing the HBV surface open reading frame. Infection media containing DMEM supplemented with 10% v/v Fetal Bovine Serum (10099, Sigma-Aldrich) and 20 mM L-glutamine (G7513, Sigma-Aldrich), 40% v/v polyethylene glycol (89510, Sigma-Aldrich) and HBV-Zeo were mixed thoroughly. Media was aspirated from the wells and the infection mix (250 mL/well) was applied to cells and left to incubate at 37°C and 5% CO_2_ for 18–20 h. The cells were washed three times with DPBS (14 040 141, Gibco), and fresh infection media was applied and incubated for 3 days. HepG2-NTCP cells were trypsinized using TrypLE Enzyme (12604021, ThermoFisher), re-seeded into 6-well plates and incubated for approximately 25 days to select for reporter HBV-infected cells, using 0.5 mg/mL Zeocin Selection Reagent (R25001, Invitrogen). As cccDNA is cleared through mitosis [51], cells in the expanded colonies eliminate cccDNA, leaving only those with integrations. The HBV integration-containing clones were expanded and frozen down until further analysis. In total, 118 single cell-derived clones were isolated.

### DNA extraction and invPCR

Total cellular DNA was extracted from cell pellets of HepG2-NTCP and HepG2-NTCP HBV-Zeo clones using the QIAGEN DNeasy Blood and Tissue Kit (69506, Germany) according to the manufacturer’s instructions and eluted in 50 μL of elution buffer. For clinical samples, total DNA was either extracted from three liver tissue fragments (5–10 mg) from each patient, using the QIAGEN DNeasy Blood and Tissue Kit, or from one FNA pass, using QIAGEN AllPrep Kit (80004, Germany). DNA concentration was quantified using NanoDrop™ 2000 spectrophotometer and stored at −30°C for further experiments. Total cellular DNA was analyzed for integrated HBV DNA using invPCR, as previously described [48]. The integration rate was determined by dividing the number of virus-cell junctions detected at the highest dilution by the number of cell equivalents within that dilution of DNA template.

Low molecular weight HBV replicative intermediate DNA was suspected of producing artefacts during invPCR. To test this, high molecular weight genomic DNA (≥10–20 kbp) was isolated through agarose gel purification. 1–5 µg of total liver DNA extract was separated by electrophoresis through a 1% low melt agarose gel (161-3111, Biorad) at 60 V for 2 h [27]. High molecular weight genomic DNA was excised with a disposable plastic drinking straw and placed in 1 mL New England Biolabs (NEB) CutSmart buffer (B6004) with 0.1% Triton X-100 (HFH10, Invitrogen). The agarose was equilibrated with CutSmart buffer at 4°C overnight. The supernatant was aspirated and 5 U *Nco*l (R0193, NEB) was added. The high molecular weight DNA was digested with incubation at 37°C for 1 h, then heat inactivated at 70°C for 20 min. The DNA was then purified using Qiagen Quick PCR Purification Kit (28104), as per the manufacturer's protocol. invPCR was then continued as previously described.

### Quantification of integrated HBV DNA by dd-qinvPCR

10 μL of the DNA extract (∼1 μg) was digested in a 40 μL restriction digestion reaction containing 1 × CutSmart buffer (B6004, NEB) and 10 U *Nco*l (R0193, NEB). The combined restriction digestion and exonuclease reaction was incubated for 60 min at 37°C. The enzymes were then heat-inactivated at 80°C for 20 min. To circularize the digested fragments, a 400 μL solution containing 500 U of T4 DNA Ligase (M0202, NEB) in 1 × T4 DNA ligase buffer and was added to the reaction. The reaction was incubated at room temperature for 2 h, followed by an inactivation step of 70°C for 20 min. Then, 10 μL of sodium dodecyl sulphate (10% w/v) was added to ensure complete inactivation of the T4 ligase. NaCl (final concentration of 100 mM) and 0.5 μL GlycoBlue (AM9516, Invitrogen) were added to aid DNA precipitation, followed by 900 μL of 100% ethanol. DNA was precipitated at −20°C overnight. The precipitated DNA was pelleted by centrifugation (14,000 × g for 15 min), washed with 70% ethanol, and air-dried. The DNA pellet was then dissolved in 20 μL of H_2_O. The final linearization step was performed by adding 20 μL solution containing 5 U *BsiHKAI* (R0570, NEB), 5 U *SphI-HF* (R3182, NEB) in 1 × CutSmart buffer and incubating at 37°C for 1 h, 65°C for 1 h, then storing at 20°C until further use.

For ddPCR analysis, 5 μL of the inverted product was put in a 20 μL ddPCR reaction composed of 1 × ddPCR Supermix for Probes (1863010, Biorad), the forward and reverse primers and the probe sequences to quantify the Integrated HBV DNA fragment were listed in Table 2. Droplets were generated according to the manufacturer’s protocol using a QX200 Droplet Generator (Biorad). Intra-droplet PCR was carried out using the following protocol: an initial 10 min denaturation, enzyme activation and droplet stabilization step at 95°C, followed by 40 cycles of a 10 s denaturation step at 95°C, a 30 s annealing step at 54°C and a 120 s elongation step at 68°C, finished with a 10 min enzyme deactivation step at 95°C. Products were then stored at 4°C until droplet reading using a QX200 Droplet Reader (Biorad), quantification using fluorescein amidite (FAM) and hexachloro-fluorescein (HEX) channels, and data analysis using QuantaSoft (Biorad).

**Table 2.** PCR primers and Probes used in dd-qinvPCR design.

Forward primer		Reverse primer	
*Sequence (5′*→ *3′)*^a^	*Position on HBV*^b^	*Sequence (5′*→ *3′)*^a^	*Position on HBV*^b^
TTCGCTTCACCTCTGCACG	1585–1603	AAAGGACGTCCCGCGCAG	1422–1405
Probes			
*Sequence (5′*→ *3′)*			*Position on HBV*^b^
/56-FAM/CCATGGCTG/ZEN/CTAGGCTGTG/3IABkFQ/			1372–1390
/5HEX/TGCTCGCAGCMGGTCTGG/3BHQ_1/			1296–1313
/5HEX/AGGCACAGCTTGGAGGCTT/3BHQ_1/			1866–1884
/5HEX/CTAGCAGCCATGGTGCTGGT/3BHQ_1/			1805–1816

^a^ The PCR primers were previously used [48] to amplify virus-cell junctions by nested PCR.

^b^ Restriction enzyme sites were predicted based on GenBank Accession #AB241115.

### Statistics

Data were analysed using the Mann–Whitney test, ratio paired t-test, or linear regression analysis in Graphpad Prism. A *p*-value of <0.05 was considered statistically significant.

## Results

### Principles of the dd-qinvPCR assay

**Figure 1.**
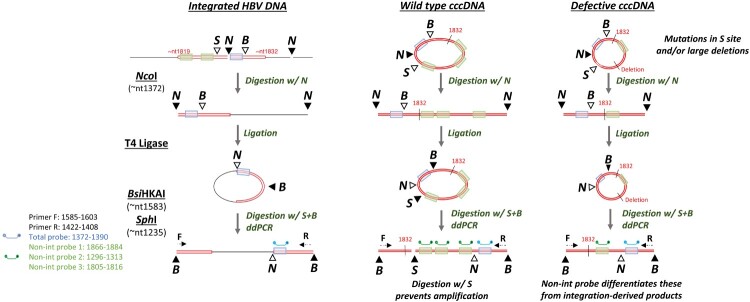
(A) Schematic diagram of dd-qinvPCR assay HBV cccDNA and integrated HBV DNA sequences (red) and cellular sequences (black) during enzymatic processing over the dd-qinvPCR protocol is shown schematically. The key HBV sequence between nt1372-nt1390 (highlighted in blue), nt1296-nt1313, nt1805-nt1816 and nt1866-nt1884 (highlighted in green). For integrated HBV DNA, the 3’ (right-hand) viral-cellular junction is excised by *Ncol* (N). Right-hand junction undergoes a T4 DNA ligation reaction, allowing DNA circularization. Circularized product is then cleaved by *BsiHKAI* (B) to form an inverted product, with viral sequences flanking cellular sequences. Finally, inverted products are cleaved by *SphI* (S) to minimize cccDNA-derived products. For wild-type cccDNA, the S site prevents the inverted HBV DNA amplifiable due to a double-stranded DNA break. A minority of defective cccDNA (contain mutations in S site and/or large deletions, shown as dashed lines) are consequently lack the S site, which allows the inverted HBV DNA amplifiable. The inverted product is then quantified using ddPCR and its forward primer (F) and reverse primer (R). The probe (blue) binds to sequences (highlighted in blue) that exist in both integrated and cccDNA forms; the probes (green) bind to sequences only present in cccDNA form (absent in integrated forms, highlighted in green). RE sites are shown as triangles (solid colouring indicates the reaction at this step); RE digestions during the steps are indicated as arrows; nt: nucleotide. Figure adapted from [21], based on the nucleotide numbering of the HBV DNA sequence outlined in GenBank accession number U95551.1. (B). ddPCR reading use drop-off assay to exclude the products with HBV sequences not expected in integrated DNA. The fluorophore FAM is used to label total probe which binds to the sequences existing in both integrated and cccDNA forms, HEX is used to label non-integrated probes which only bind to the sequences present in cccDNA forms, allowing the simultaneous detection of two different targets in the same reaction. Orange points indicate cccDNA amplicon, and blue points represent HepG2-NTCP Zeo clones. Y-axis represents the FAM signal amplitude (the total HBV DNA probe); X-axis represents the HEX signal amplitude (the cccDNA specific probe).

We developed a novel method to expedite the quantification of virus-cell junctions using a ddPCR-dependent approach (Figure 1). Inversion first occurs as per previously published approaches [22,23,47,48]. The genomic DNA is first digested with *Nco*l that cuts at known sites within the HBV genome. The resulting DNA fragments are then then added to a T4 DNA ligase mediated ligation reaction in a large volume to promote intra-molecular DNA circularization (as opposed to inter-molecular joining of DNA fragments). The circularized product is subsequently cleaved with *BsiHKA*I to form an inverted product, with viral sequences flanking unknown cellular sequences. The inverted products are also cleaved with *Sph*I to reduce aberrant amplification of HBV cccDNA.

Products are amplified using specific HBV-specific forward and reverse primers [48], which are oriented in a way that they amplify across the junction of the integrated viral and host DNA. The total HBV DNA probe (FAM) binds to sequences that exist in both integrated and cccDNA forms, while “non-integrated” probes (HEX) only bind to the sequences present in cccDNA forms (due to being outside of the expected excised HBV DNA junction derived from double stranded linear forms). Multiple “non-integrated” probes were used to detect the broad range of products derived from defective cccDNA templates sequenced by invPCR in our previous studies [22,23,47,48]. The ddPCR drop-off assay differentiated cccDNA from integrated DNA derived products based on HBV sequences not expected in integrated DNA.

### Sensitivity, linearity and specificity of the dd-qinvPCR assay

**Figure 2.**
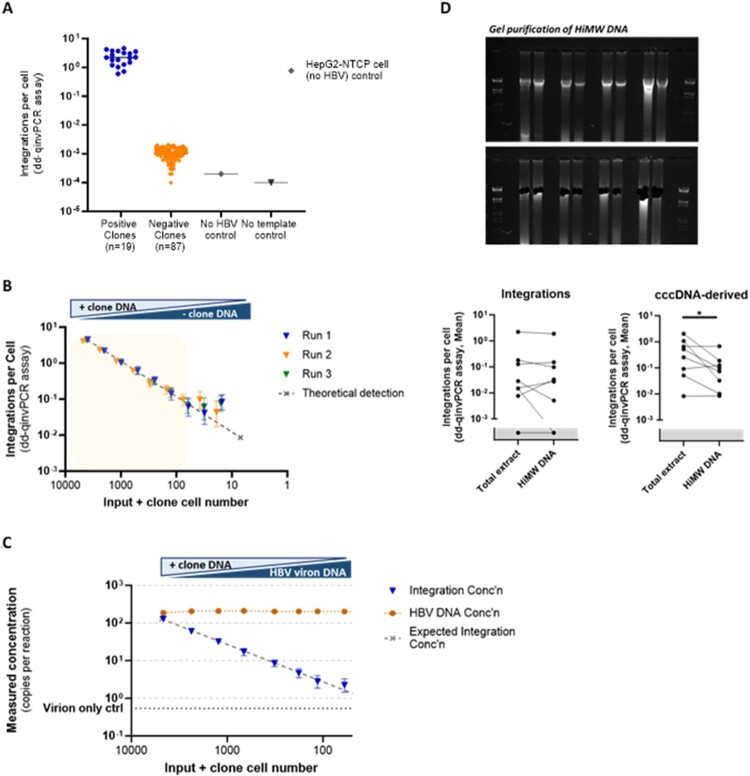
The sensitivity and specificity for dd-qinvPCR assay to quantify integrated HBV DNA. (A) The sensitivity of dd-qinvPCR assay integrated HBV DNA was quantified in DNA extracted from 106 HepG2-NTCP Zeo clones by dd-qinvPCR, these were performed in duplex with reference gene RNaseP (2 copies/cell) for normalization. Inverted DNA that tested positive for integrations among 19 clones (blue points), and significantly above 87 negative clones, uninfected HepG2-NTCP cells (no HBV) and no template control. (B) The linearity of dd-qinvPCR assay was determined by titrating down the positive clone DNA input from 1 μg to 4 nanogram (ng), while DNA from a negative clone was increased to maintain constant DNA input. The integration rates of three repeated runs (triangles in blue, orange and green), the expected integration rate per serial dilution is shown as dashed line, X-axis shows the equivalent input positive clone cell number (C). The specificity of dd-qinvPCR assay for integrated HBV DNA in relation to HBV DNA replicative intermediates DNA from a positive clone was titrated from 1 μg to 4 ng, while supplemented with virion-derived HBV DNA to maintain constant HBV DNA integration rates. dd-qinvPCR was used to quantify the integrated HBV DNA concentration (blue triangles) and total HBV DNA concentration (orange squares). X-axis shows the equivalent input positive clone cell number. The expected concentration (in absolute copies per microliter) assuming 100% detection efficiency is shown as dashed line. False positive signals for integration came up at ratios of 1 copy per ∼300 copies of virion DNA (virion only control in dash line). (D) The specificity of dd-qinvPCR assay by analysing both total and high molecular weight (HiMW) DNA. HiMW DNA was isolated through agarose gel electrophoresis of 1–5 μg of total liver DNA extract. Using dd-qinvPCR assay to quantify the integration rates of HBV DNA in paired total and HiMW preparations of the same DNA extract, there was no significant difference (left, *p* = 0.349, ratio paired t-test); the HiMW DNA samples showed ∼10 times lower integration rates of cccDNA (right, *p* = 0.032, ratio paired t-test).

Each HBV DNA integration event occurs at a random site in the human genome [21]. Due to the distribution of restriction enzyme sites used for the inversion and efficiency of circularization of the excised chimeric DNA fragment, not all integrations are detected. Our previous modelling suggested that invPCR could detect ∼10% of all HBV DNA integrations [21]. We empirically tested this assumption using 106 single-cell-derived clones containing unique integrations. These were generated by infecting HepG2-NTCP cells with a reporter HBV virus (encoding a Zeocin resistance gene in place of the surface open reading frame). Cells with HBV DNA integrations were selected for using Zeocin and resultant colonies were isolated for DNA analysis.

dd-qinvPCR reactions with DNA from parental cells and no template reactions were negative as expected. By analysing this bank of clones with single integrations, we detected viral-cellular junction in 19 of 106 clones (18%) (Figure 2A), relatively consistent with the *in-silico* estimations [21]. Thus, the actual integration rate is likely ∼5 times higher than what is detected by the invPCR and dd-qinvPCR assays.

DNA from these positive clones were analysed by standard invPCR, and products were detectable in all of them (and not negative clone controls). We were able to isolate PCR products from DNA agarose gels for 13 clones and sequence them by Sanger sequencing, confirming that they are true integrations (the sequence of human-HBV junction of the 13 clones provided in Supplementary Table 1).

We then tested the linearity of the assay by titrating down the input of DNA from a detectable clone (clone 1, the sequence of human-HBV junction in Supplementary Table 2), while keeping the total input constant with a non-detectable clone (clone 14, the sequence of human-HBV junction provided in Supplementary Table 2) (Figure 2B). We found that the dd-qinvPCR assay could detect integrated HBV DNA in a linear fashion and hewed closely to the expected theoretical value, down to ∼60 cell equivalents in an input of 4800 cells per inversion reaction.

Moreover, we measured the specificity of the dd-qinvPCR assay and its ability to discriminate integrated HBV DNA from other HBV DNA forms (Figure 2C). To test this, we mixed DNA extracted from heparin-affinity purified HBV with DNA from a cell clone that was dd-qinvPCR positive (clone 1). We altered the ratio of these two sources, maintaining a constant copy number of total HBV DNA per reaction. Consistent with the previously determined lower limit of detection, we found linear quantification of the integrated forms down to an input of 72 cells per inversion reaction. The virion only control showed a false positive rate of <1 copy of integrated HBV DNA per 300 copies of HBV DNA.

We then determined if the HBV replicative intermediate DNA within total cellular DNA extracts was at an integration rate sufficient to produce false-positive artefacts. In separate patient samples, we specifically isolated and analysed high molecular weight genomic DNA (thereby depleting intracellular single and double stranded HBV DNA). When we compared the dd-qinvPCR assay analysis of paired total and high molecular weight samples from the same tissue, no significant difference in integration rate was observed (Figure 2D). In contrast (and as expected), significantly lower integration rates of cccDNA-derived forms (∼10 fold) were observed by the dd-qinvPCR assay in the high-molecular weight DNA extracts. Thus, we show that our assay is likely to be highly specific for integrated HBV DNA and that the impact of intracellular replicative intermediates on the signal is minimal.

### HBsAg-loss is associated with significantly lower total HBV DNA integration rates and fewer integrations

We then analysed intrahepatic total HBV DNA and integrated HBV DNA integration rates in liver tissue from people with chronic HBV infection (n = 18) or those who had cleared HBsAg (i.e. functional cure, n = 15). Three fragments of liver tissue (non-tumour) from each patient were analysed by ddPCR for total HBV DNA and by dd-qinvPCR for integrated HBV DNA. We also quantified integration rates in these tissues using our previously published assay (invPCR) and found high correlation between the two methods (r^2^ = 0.0228, Supplemental Figure 1). However, many samples positive for dd-qinvPCR were not positive using invPCR. The likely cause for this is the necessitation that integrated forms must be more frequent than the defective cccDNA forms to be detected by invPCR (due to its dependence on end-point titration to isolate single-copy templates for nested PCR). Discrepancies between the quantification of integrations by invPCR and dd-qinvPCR may also be due to the inherent poor precision of end-point titration for quantification. Given these likely explanations, we suggest that the figures provided by dd-qinvPCR are more accurate than those from invPCR.

**Figure 3.**
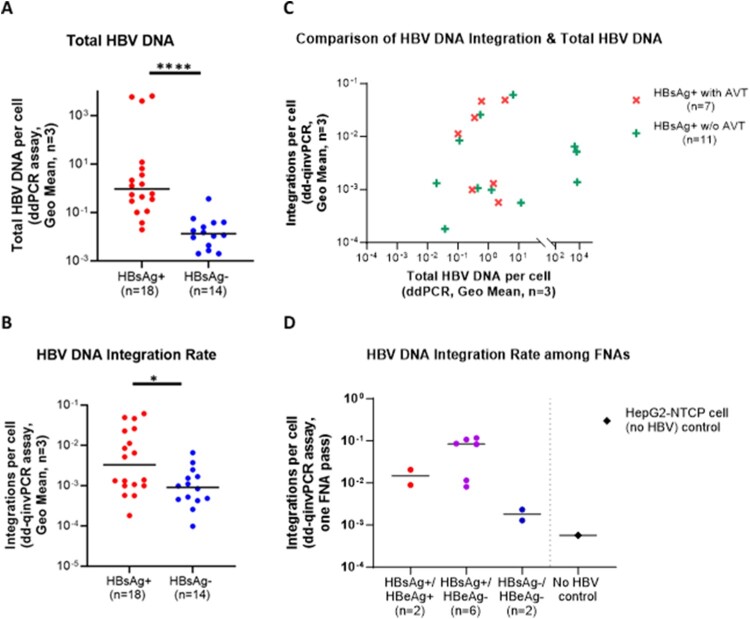
Intrahepatic total HBV DNA and integration rates in patients. (A) Quantified total HBV DNA in HBsAg-positive (n = 18) or HBsAg-negative (n = 14, undergone functional cure) by ddPCR, significantly lower integration rates (*p* < 0.0001) of total HBV DNA were observed in the HBsAg-loss group compared to those with chronic HBV infection. (3) Quantified integration rates using dd-qinvPCR, the integrated HBV DNA in HBsAg loss group (n = 14) was significantly lower (*p* = 0.0179) than active HBV infection (n = 18). (C) Comparison of intrahepatic copies of integrated HBV DNA per cell and total HBV qPCR among HBsAg-positive patients that under antiviral treatment (n = 7) or those without treatment (n = 11). No significant association was observed between total HBV DNA and the integration rates of integrated HBV DNA in antiviral treatment group (r^2 ^= 0.1083; *p* = 0.471), or treatment naïve group (r^2 ^= 0.0447; *p* = 0.533). HBsAg-positive patients under antiviral treatment did not have significantly different integration rates of HBV DNA integrations compared to those not under treatment (1.90 × 10^−2 ^± 2.13 × 10^−2^ vs 1.03 × 10^−2 ^± 1.85 × 10^−2^, *p* = 0.479). Integrations per cell and total HBV DNA represent the Geometric Mean of those 3 fragments. (D) Comparison of copies of integrated HBV DNA per cell in patient FNAs among 10 patients underwent FNA, 2 patients are active hepatitis B infection (HBsAg-positive and HBeAg-positive), 6 patients are chronic hepatitis B infection (HBsAg-positive and HBeAg-negative), 2 patients are no active hepatitis B infection (HBsAg-negative and HBeAg-negative). The copies of detected integrated DNA per cell are 5-fold higher (median 0.8431 vs 0.01478) in chronic infection patients (n = 6) than active infection (n = 2). Compared with uninfected HepG2-NTCP cells (no HBV), the integrated HBV DNA in HBsAg-negative patients (n = 2) is detectable and significantly lower (*p* = 0.0444, Mann–Whitney test) than HBsAg-positive(n = 8) group.

As expected, total HBV DNA copies per cell were significantly lower in the HBsAg-loss group compared to those with chronic HBV infection (Figure 3A; *p* < 0.0001, Mann–Whitney test), which is consistent with prior studies [52,53]. Importantly, the integration rate in the HBsAg loss group was significantly lower than in patients with active HBV infection (mean ± SD of 1.514 × 10^−3 ^± 1.839 × 10^−3^ vs. 1.365 × 10^−2 ^± 1.986 × 10^−2^ integrations per cell; *p* = 0.0179, Mann–Whitney test), supporting our hypothesis and indicating that natural clearance of HBV is associated with fewer viral integrations (Figure 3B).

In HBsAg-positive people, there was no significant association between total HBV DNA and integration rate (*p* = 0.375) (Figure 3C). Moreover, HBsAg-positive patients receiving antiviral treatment did not have significantly different integration rates compared to those not receiving treatment (1.90 × 10^−2 ^± 2.13 × 10^−2^ vs 1.03 × 10^−2 ^± 1.85 × 10^−2^, *p* = 0.479, Mann–Whitney test). This shows that replicative intermediates were not likely to be interfering with the quantification of integrated HBV DNA using our assay.

As our assay displayed high sensitivity and required only low DNA input (Figure 2C), we determined if HBV DNA integrations could be quantified in clinical FNA samples (Figure 3D). Liver FNA is a minimally invasive procedure used to obtain tissue samples, offering a balance between diagnostic accuracy and patient safety [54]. It has been demonstrated to be a reliable method for obtaining serial hepatic tissue samples and can obtain small numbers of liver cells (10^4^–10^5^ cells) [55]. By analysing DNA extracted from 10 FNA samples, we found that dd-qinvPCR could quantify integrations rates that were similar to those observed in liver resection tissues. Two of the patients were HBsAg-negative and had lower integration rates compared to patients who were HBsAg-positive.

### Integrated HBV DNA is not associated with other clinical features

**Figure 4.**
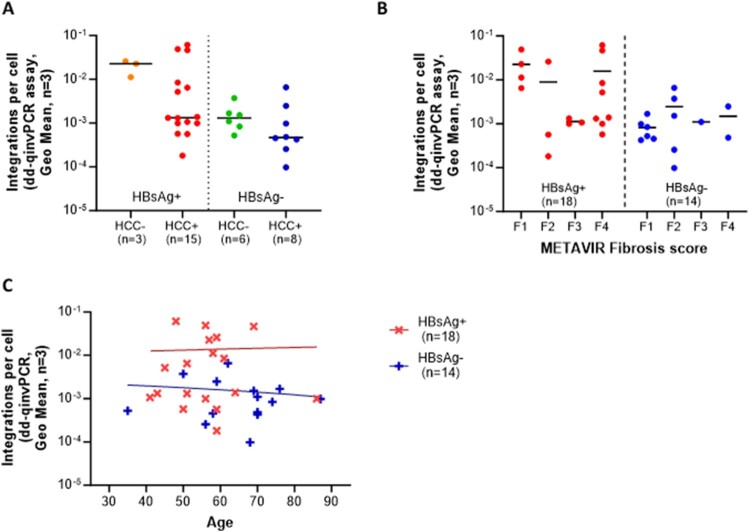
Intrahepatic integrated HBV DNA integration rates among patients with different clinical features. (A) Integration vs. HCC no significant difference in integration rate was observed (*p* = 0.246) between HCC + (n = 23) and HCC− groups (n = 9). (B) Integration vs. fibrosis stage no correlation with fibrosis stage in HBsAg-positive (n = 18) and HBsAg-negative (n = 14) groups. Fibrosis grades were classified using the METAVIR scoring system [49] determined by liver histology: F1 means portal fibrosis without septa, F2 means few septa, F3 means numerous septa without cirrhosis, F4 means cirrhosis. (C) Integration vs. age no significant relationship between age and integration rates were observed by linear regression in HBsAg-positive (n = 18, *p* = 0.888) or HBsAg-negative (n = 14, *p* = 0.648) cohorts. Integrations per cell are represented by the geometric mean of 3 liver fragments per patient.

We performed subgroup analyses to examine whether integration rates were modified by clinical features (HCC and cirrhosis) or age. We found that integration rates were not statistically associated with the incidence of HCC (Figure 4A), fibrosis stage (Figure 4B), or age (Figure 4C) in these HBeAg-negative cohorts.

## Discussion

In this study, we established a highly specific, sensitive, and precise method (dd-qinvPCR) to detect and quantify HBV DNA integrations, even in challenging and limited samples such as FNAs. Using this assay, we found that HBsAg loss (but not other demographic or clinical features) in HBeAg-negative patients is linked with lower integration rates.

The underlying mechanism of this association is not known, as we lack key clinical data to make this distinction (e.g. the time of seroconversion relative to surgery). On one hand, if clearance of HBsAg occurred in the distant past (i.e. decades prior), this result might be due to the reduction in ongoing integration events. Moreover, the decreased inflammation from clearance would reduce the clonal expansion of hepatocytes that is known to drive integration rate increases (e.g. due to HBeAg seroconversion) [27,56]. On the other hand, if HBsAg-seroconversion occurred in the recent past, then this would suggest that the reduction of integrated HBV DNA is necessary to induce HBsAg loss or is caused by the antiviral immune response associated with it (e.g. clearance of HBsAg-producing cells). Distinguishing between these two possibilities will be difficult, given the general lack of clinical justification to perform liver biopsies in patients who have cleared HBsAg. However, our novel assay provides one potential approach: the high sensitivity of dd-qinvPCR allows it to be used to quantify integrations from liver fine needle aspirates, which are less invasive than core needle biopsies.

Nevertheless, we found that HBV DNA integrations in HBsAg-negative patients were not completely eliminated and were still detectable, consistent with animal models [20] and clinical data [57,58]. Our approach was not able to define the full sequence of integrations remaining in the liver after HBsAg loss. Defining the structures of the persisting integrations (e.g. whether they maintain the HBsAg open reading frame) or their epigenetic state (e.g. whether they have been transcriptionally silenced) could provide understanding of the intrahepatic landscape after functional cure of chronic HBV infection and inform therapeutic approaches to induce it.

While several recent studies [59–64] showed nucleoside analogue treatment may reduce several aspects associated with integrated HBV DNA over time (e.g. transcriptionally active integrations), our data did not support the reduction of integrated HBV DNA with antiviral treatment. Reductions of integrated HBV DNA with antiviral treatment have observed in previous studies, but they are only limited changes (∼1 log) over long periods of time (10 years) [60]. It is possible that our small retrospective study was not powered enough to show these small differences. Moreover, the clinical information from the participants in the current study was not detailed enough to provide the length of antiviral treatment prior to surgery, limiting further interpretation over the effect of antiviral therapy on integration rates.

Nonetheless, we believe it is important to understand the impact of new curative therapeutics on HBV DNA integration. As integrations have been shown to be major contributors to HBsAg secretion in HBeAg-negative patients, if reductions of HBV DNA integrations can be achieved, such approaches may be valuable to induce functional cure, in combination with other agents. In particular, quantifying integrations before and after immunotherapies could help understand whether integrations are specifically targeted by therapeutically-activated immune responses. Given that our dd-qinvPCR assay appears to be one to the most sensitive and quantitative approaches available, we believe that it could facilitate this analysis by providing a way to quantify HBV DNA integrations from liver fine needle aspirates (which could be incorporated into clinical trial monitoring).

However, this assay does have limitations. Not only does it detect only a proportion (∼18%) of large HBV DNA integrations (i.e. containing enough of the HBV DNA to include the zeocin selection cassette replacing the HBsAg ORF), but it is likely to miss integrations with large terminal deletions (due to missing primer binding sites). It is still unclear to what extent these sorts of integrations contribute to carcinogenesis or HBsAg expression.

In summary, our work provides a new approach to study HBV DNA integrations and shows that HBsAg loss is associated with fewer integrations. These results highlight the potential importance of targeting HBV DNA integrations with curative therapies and provide a tool to quantify them. Our novel method could be used to inform future HBV cure research, which would benefit the 300 million people worldwide living with chronic hepatitis B.

## Supplementary Material

Table S1.pdf

Table S2.pdf

Figure_S1.TIF
