# Supplementary material for: Novel digital droplet inverse PCR assay shows that natural clearance of hepatitis B infection is associated with fewer viral integrations
Source: Emerg Microbes Infect. 2025 Jan 3;14(1):2450025. doi: 10.1080/22221751.2025.2450025 (PMC11731057; doi:10.1080/22221751.2025.2450025)
Supplement: Table S1.pdf [file TEMI_A_2450025_SM0041.pdf]

Supplemental Table 1. The Sequence of Human-HBV Junction in 13 clones

Clone	HBV junction	Chromosome	Sequence *
1	1821	17	*CCAAATATTGCCCAAGGTCTTACATAAGAGGACTCTTGGACTCTCAGCAATGTCAACGACCGACCTTGAGGCATACTTCAAAGACTG<br>TTTGTTTAAAGACTGGGAGGAGTTGGGGGAGGAGATTAGGTTAAAGGTCTTTGTACTAGGAGGCTGTAGGCATAAATTGGTCTGC<br>GCACCAGCACCATGCAATATATTGTAAATATTTTTGCTTGTTTGTAATCTTGTCTTTTAACTTTATGTTGACTTTTACCATTCAGATGCA<br>AAAAAATTGAAGTAGTTAAATTTATCAGTGTTTTTCCTTTATGACTTATGGGATTTGTCTTATTCTTAGATGTTCCCATATGGTGTATT<br>CCTATACGGGGTGTGTGTGTGTGTGTGTGTGTCTGTGTGTGTGTGTAATTTTTTTTTTTT*
2	1816	10	*GCCACCAAATATTGCCCAAGGTCTTACATAAGAGGACTCTTGGACTCTCAGCAATGTCAACGACCGACCTTGAGGCATACTTCAAAG<br>ACTGTTTGTTTAAAGACTGGGAGGAGTTGGGGGAGGAGATTAGGTTAAAGGTCTTTGTACTAGGAGGCTGTAGGCATAAATTGGT<br>CTGCGCACCAGCACCAGAGTAAATCTATGTCTCTAACAGACATGGTCAACCTGCTTCCCCATTTCCCATACAGTACTCCTCCCCTGGGT<br>CATGCTCTCCCACCTGAGGGTCCGTGTGTAGTTCAGAAAGGGTGTGATACCCAGGAACTTCTGGATGCTCTCCATTGAGGCTGCTGG<br>GTTGGTACGCAGCTCTTGCCCATCCACAATCAGCAACTAAAAGGACAGGGATGTGGTTCCCTCTATATTCCTAAGAAGCCTCTGGGC<br>TGAAGGGAAGAAGAGGGGAAGGGGAACCCCACTGAGGGCTCTTGTGGGGATTATACCTGTCCAGAGGGGTAGTAAGTCAGCCAG<br>CGTTGTAGATGGGTAGAATAGTAGCCAGGGACAAGACAGCGGTTCTGCAGGGAGCGTAGTGCCAGAGGGGTCTGGGAGGAGGCT<br>GAAATCACCTGATAGAAGGTATAGTTCAGAGCAACTGGGTCTCCATGGCTGCTAGGCTGTGCTGCCAACTGGATCCTGCGCGGA*
3	1818	20	*CCAATATTGCCCAAGGTCTTACATAAGAGGACTCTTGGACTCTCAGCAATGTCAACGACCGACCTTGAGGCATACTTCAAAGACTGT<br>TTGTTTAAAGACTGGGAGGAGTTGGGGGAGGAGATTAGGTTAAAGGTCTTTGTACTAGGAGGCTGTAGGCATAAATTGGTCTGCG<br>CACCAGCACCATGAGTGCAATGGTGTGATCTTGGCTCACTGCAAACTCCGCCTACCAGGTTCAAGCGATTCTCCTGCCTTAGCCTCCC<br>ACATAGCTGGGATTACAGGCTTCGAAGCCACTTTTAGAGATAAGCAGATATGGCATCTAAATATTTCACTACTGTAATAAGTTAAAA<br>AATAACGCAGATGGAGTCAAAAGTAAATAAAATACACCACTCAGCTTTGGAGACAGCAGCTAGAAATGTCCAGCACCAGCAGAACC<br>GTGCAATGCCAGGATGGTGTCTACCCCACAGGTTCCAGGTCAGCCAAGAAGAGCATGGGACACCCAGGGGACCTGCCTGACAGGG<br>GCTGCAAACTCTCACTGGCTACAGCTAGAGAGACATGGAGGGCGAACCTCAGGGTCACGGGCACGAGAGGGGCATCAGGGAACC<br>AAGGGAAGAGGGTGGTTGGAGAGCAGAGGAACCGGGTGGCGAGGGGGACCCCTCAAGAGAAGCCGCCCACCCCGGCCTGAGGC<br>CCACCCTAGAAGCATAGCCAACGAAGCCCAGGCTCCAGCTCCCTTATCCAGCTGAGACCCCTCTCGGCTGTTGCCCCTGGCCATCTTG<br>GCCCACCTGGCAGCTCCCGGTCAAGCCCAGGAGCCTTTATTATTTATCTGACTTTCCTGATTTTCTGGCTCCCATGGCTGCTAGGCTGT<br>GCTGCCAACTGGATCCTGCGCGGGACGTCCTT*

4	1830	11	CCAAATATTGCCCAAGGTCTTACATAAGAGGACTCTTGGACTCTCAGCAATGTCAACGACCGACCTTGAGGCATACTTCAAAGACTG<br>TTTGTTTAAAGACTGGGAGGAGTTGGGGGAGGAGATTAGGTTAAAGGTCTTTGTACTAGGAGGCTGTAGGCATAAATTGGTCTGC<br>GCACCAGCACCATGCAACTTTTTCTCCCTGGTCCCCTAGAGTCTGGAACATAATGGATTGTATCCTCCTGGGTCTGCTTATTTTCCTGC<br>GTTAGGAACTCTCCCAATTCCTTTGAACATTCTTCATACACGAACATTCCTCATCGGACAACTGGGTTATGGTCTGAAGTCTATCATGT<br>CAGGGCAAGTCACTTATCCCCACTGAGCCTCCAGTTCTCCATCAGTAAAAATGAAGAAACGAATACTATCTCATGGGTAAAGATTAG<br>CTTGGAAAAGTAATATGAAAGTTTAGGGTTTGGAAGAGACACTTGGTAAGTATTAAAACAATTTTTTTAAATCACAGTTTTGAAATA<br>GGAGTTAGTAGGTAAGCCCATCTGGTATTGCCCTTAGCTCTTTATAACACTTCCAAAGGAAATCATTCCAGAAAGTCAGTGCCACTCC<br>TGGCTTCAGAACGGTGGCAAATCAGGTAGAGGATGCCCTCCTATATGCATAAACGAAATCCCCAGGGTCTGACTTCTTCTGTGAAG<br>AGCCTCCTAAGTTCTAGGCTCACTCACTTATCTTATTCTGCAGGCCAGCACCAAGGCCCAGAGAAGACCCACTAGATCCATGGCTGCT<br>AGGCTGTGCTGCCAACTGGATCCTGCGCGGGACGTCCTTTAA
5	1828	2	AACGCCACCAAATATTGCCCAAGGTCTTACATAAGAGGACTCTTGGACTCTCAGCAATGTCAACGACCGACCTTGAGGCATACTTCA<br>AAGACTGTTTGTTTAAAGACTGGGAGGAGTTGGGGGAGGAGATTAGGTTAAAGGTCTTTGTACTAGGAGGCTGTAGGCATAAATT<br>GGTCTGCGCACCAGCACCATGCAACTTTTTCCAGAGTGAGGATCTGTCAAAAAAAAATAAATAAATAAAAATAAAAATAAATCATTGG<br>CTTTCACAAAACAGAAGTAGGGATTTCATGAACAATTTCCGAGTTATAATCATAACAAAGGCCACTGGCCATGTGTAGTCTTCTGTT<br>GTATGTGTCACAACCATGGCTGCTAGGCTGTGCTGCCAACTGGATCCTGCGCGGGACGTCCTT
6	1829	2	GCCCACCAAATATTGCCCAAGGTCTTACATAAGAGGACTCTTGGACTCTCAGCAATGTCAACGACCGACCTTGAGGCATACTTCAAA<br>GACTGTTTGTTTAAAGACTGGGAGGAGTTGGGGGAGGAGATTAGGTTAAAGGTCTTTGTACTAGGAGGCTGTAGGCATAAATTGG<br>TCTGCGCACCAGCACCATGCAACTTTTTCAAATGTTCTTACATCATATGGTCTAGAACAAGAAAATAATAATAAACAGATGATTGGG<br>CATTTCCTGAGTTGAATTTTTTCGATTTGGTACAATTAAAATAACACAGTGTGGTAATGCTACTGATATTTCTAAAGCCACCACCACCA<br>CTTGTATTGAGTGCTTAATGTATACTGTTGACTTTGCTAGACCTTGTTACATACATGTTCTACAACTCCATAAGGTAGATGGTGGTAT<br>CTCTATTATACAGATAGGAACACTAAAGCATGGACAGGTTAAGTATCTCCTCAAAGGCCATGTAGCTGAGAGGTTAAAAAAAATTTA<br>CTAGGCCAAAAAAAATTGATATAAGGCATAGAAAAAAATTTAAGAAGACTTTTAACATAAGGAGGAAATAAAAAGGACTGGCTGC<br>CTTGCAGCAGCTCCTAAAACCTAAAGCTGAAAAGAAAACTATAAATAGGTGAACTTGTCCTATTTCCTCCAGATTTTTGAATTATGTA<br>TTTTTGCATGAGTGTAATTCTCATATGCAGAAGAATAGATAGCTTAGCATAGTACTTATTATTTGTTGAAGAAGCATTGGAATGTGG<br>CTGTGGTGATTGGAGGAGATGAGGTCTGAGATGTGTGAAGGACCAGATCATGTAGGGTCTCTGGAGGCCACATTAAGGACATTGA<br>GTAAGATGGGAAGATGGAGGAGTAGGGTTTTAAGCAACAAACTGACATGATCTGACCTATAATTTATAAAGTTCATCCTAGTTGGA<br>GCCTGAAGAATTTATTTGGGTGGTGCAGAATAGAAGGAGGCCCCTAGTAAGGGCGTTGGCTATTGAAAGTCCAGGAAGAGCAATG<br>GTGGGGTAGGAATAAAGTGGGAATGGTGGAAGTGTTCCCTTCCACGAAGGACTCACCTCCTAGTCA

7	1800	6	*GCCCACCAAATATTGCCCAAGGTCTTACATAAGAGGACTCTTGGACTCTCAGCAATGTCAACGACCGACCTTGAGGCATACTTCAAA<br>GACTGTTTGTTTAAAGACTGGGAGGAGTTGGGGGAGGAGATTAGGTTAAAGGTCTTTGTACTAGGAGGCTGTAGGCATAAATTGG<br>TCTTTATAAAAATCCTTAAAATGAGATGTTTGCCTGTCCTAAGAACTTACTGACCCAACACCTGATACATACAGGTTACCCAAGCAAA<br>TCAGAGAAGCTCTGGAGCATGGAAAAGATGAAAACCTATTTCTGCCTCATAAGGAATCTGCAGCCCACACTGTCTGATGTGGGCAA<br>TCAGGTTCTTCTCCGGTACTACCAGATGCAAAGGCAGAGTGATTGCCGGAACGCTGCCCGGACCACCATTCGGCTGTTGGAAAGCTT<br>GATACGATTAGCAGAAGGTCTATTTCATTCAGCGAATGATGCTTGTATTGAATGCTGCGTGTGTGCAAGGCATGGGGCTTGGCACC<br>ATGGCTGCTAGGCTGTGCTGCCAACTGGATCCTGCGCGGGACGTCCTT*
8	1830	5	*CCAAATATTGCCCAAGGTCTTACATAAGAGGACTCTTGGACTCTCAGCAATGTCAACGACCGACCTTGAGGCATACTTCAAAGACTG<br>TTTGTTTAAAGACTGGGAGGAGTTGGGGGAGGAGATTAGGTTAAAGGTCTTTGTACTAGGAGGCTGTAGGCATAAATTGGTCTGC<br>GCACCAGCACCATGCAACTTTTTCACAATTCACAAATATGTGGAAATTGAACATACTCTTAATAACAAATGGGTCAAAGAAAAAATA<br>AAAGAGGAAGTAAGAAAACATCTTGAGACAAATGGAAATGAAAACACATTATATCAAGCATATTCAATTCAGTAAAAGTAGTACCA<br>AGGGAGAAGTTCACAATGATTAAATGCCTACATTTAGATAATAGGAAGATCTTAAAGAAGCAACCAAATTCTATACCTCAAAGAATT<br>AGAGAAAGAAGAACAAACTAATTCCAAAGATAGCAGAGGAAAGAAAATGATAAATGTTAGAGCAGAAATAAAATAGAGACTAGA<br>AAAACAATAGAAAAAAATAAACTAAGAGTTTATAAATCTTTGGTTAAACTTAAAAAAAAGAGAAACTATTCAAATAAATAAATTATC<br>AGTAATGAAAGAGGAAACATTTCAAATGATGACCAGAGGAAAAAATAAAAGAATTATGAAACTGCTAGAGAGTGAATCAGTAATC<br>AAAAACCTCCCAAGAAACCTCCAGAACCAGATGGCTTCATTGGTGATTTCCACCAAACATTTAAAGAATAACTAACGCCAAACTTTTC<br>TAAACTTGTCCAAAATTTGAAGAGGAAAGGACACTTTTGTACTCATTTAATGAGGCCAGCTTCATCTGATACCAAAGCCATACAAAG<br>ACACTGCAAGAAAAGATAACTGTAGGCCACTATTCCTAATGAATACAACTTTAAAATTTTCACAAAATATAGCAAACTGAATCTAACA<br>GTACGTAAATGATAATACACATGGCTGCTAGGCTGTGCTGCCACTGGATCTGCCCGGG*

9	1828	2	*AACGCCACCAAATATTGCCCAAGGTCTTACATAAGAGGACTCTTGGACTCTCAGCAATGTCAACGACCGACCTTGAGGCATACTTCA<br>AAGACTGTTTGTTTAAAGACTGGGAGGAGTTGGGGGAGGAGATTAGGTTAAAGGTCTTTGTACTAGGAGGCTGTAGGCATAAATT<br>GGTCTGCGCACCAGCACCATGCAACTTTTTCCAGTTTTGCTCATAGGCACATATTTAGCAGTACTCAGCTGAAGATTAAAGGGCAAA<br>ACAATGTGGATTTTTGGCGCTCTCCTCTGTGCAATTCTCTTTGTTCAAGTACTTTCACCTGCAGTCTCTAGACAGCTTGATCTCCTTGG<br>CTAGCCAACTCTATCTCCCCAGCTCATGGAATCCACTGGACTCAGCCTGCTTCACCTCATACCCCATGCTGTGGCCTGAAAGTCAGCT<br>GGGGCAATTATAGGGCACATCTTTCTTCCTCTCTCTCTCAGTGATCTTGTGCTGCTCTGCCTGATATCGGTGTCTGAAAATCGTTGTTT<br>TGTATATTTTGTCCGGATTTATTTTCTTGTTTCAAAAAAGAGGATAAATCTAGTTCCTATTACTCCATCTTAGCCTGAAGTGGAAATAA<br>TTTCTCACTCTATTTTTCAGCCTAAGTTTTTATATGTGGATAGAGTATGTAGCAGAGTCAGAGAGTAGAAAAAAATTACAGGGAGAGT<br>AACATATCACTCCCTCAGTGTTCTCCTTTCAACTTCTGTATCCCCAGATACTATCAAAGACATCTGGGTCAAACCACATTGATTGCTGG<br>TTGTCTCTCCTTAATCAAGTCCTACATTATCTAGATCCTATTCTGTAGACTCAGCTGAAGAGAAAGAATAGTGTAGGTTCCCCCTTGCC<br>CTGCACTCATGCTTTAACATACTAAAGTGCTTTACGGTGTCAATAAAAAGAGTCAAATTCTAAAATATTTGAGGAGATTTATTCTGAG<br>CCAAATGTGAGGACAATAACCCATGGCTGCTAGGCTGTGCTGCCAACTGGATCTGCG*
10	1793	22	*GAACGCCACCAAATATTGCCCAAGGTCTTACATAAGAGGACTCTTGGACTCTCAGCAATGTCAACGACCGACCTTGAGGCATACTTC<br>AAAGACTGTTTGTTTAAAGACTGGGAGGAGTTGGGGGAGGAGATTAGGTTAAAGGTCTTTGTACTAGGAGGCTGTAGGCATAGGC<br>AGAGTAGTGATGTCAAAGCTCCTTACAAGTAGAATCGGAAACTGAAAGTCTACTGTCTTTTTTCTTTTAAACATACTTTCTACTGAAA<br>TGCATATCTTGGTATTTTATCATGTCTTACTTTGTATTTAATTTGCTGGGTGAAACGTTTGTTTCACTGGTTGGTATCTTTTGGATGTG<br>GATGCTATATTTATTTTTTAGGATTGAAGTAGATGGTGATGCTAACTCCTATATAGACACATTACGGAAATATACTAGACCAACACTG<br>CAGATGGTAAGTGTCTAGTTGATACATGTACAATCTTTTAGTTATTGTTCCAAACCATCTTGATAAGTATTTTAGAATTCAAAATGTT<br>GTTGTTGGTTTCCTGAACTTGCCAACGAAAGCAGATTAACCTGCTATTAGCTGAATACAAACTTTCATGGTTTAGCTATTCCTGAGTA<br>TCACAATCGTATGCTAATTTTCTCAGACTTGGAGACTCGTAATTACTTTTAAAATCTCCTAACTTGAAAAAATACACCATACAAATGTA<br>AAGTAGTGTGGGTATTAATAATCACATTCATTACTTTTACCCGCAGGTGTAACCTGCCAAGTCTGTGATCACGATCTTCTAGCCTATT<br>ATTCTTCTTGCCTGTTTTTTGTTGTTGTTGTTGTTTTTTGTTTGTTTGTTTGTTTTTGAGATGGAGTCTTGCTCCATGGCTGCTAGGCTG<br>TGCTGCCAACTGGATCCTGCGCGGAAACGTCCTT*
11	1827	6	*CGCCACCCAAATATTGCCCAAGGTCTTACATAAGAGGACTCTTGGACTCTCAGCAATGTCAACGACCGACCTTGAGGCATACTTCAA<br>AGACTGTTTGTTTAAAGACTGGGAGGAGTTGGGGGAGGAGATTAGGTTAAAGGTCTTTGTACTAGGAGGCTGTAGGCATAAATTG<br>GTCTGCGCACCAGCACCATGCAACTTTTTTTAAAATGGGAAAGGCCTTTCTATGGCATTAAAAATTGAGAAACCAAAAAGGTAATAT<br>AACAGGTTTGATTCAGTAGTTTGGAAACTGTCTGCATGACATGAATTACCCATGGCTGCTAGGCTGTGCTGCCAACTGGATCCTGCG<br>CGGGACGTCCTTTA*

12	1769	1	*CGCCACCAAATATTGCCCAAGGTCTTACATAAGAGGACTCTTGGACTCTCAGCAATGTCAACGACCGACCTTGAGGCATACTTCAAAGACTGTTTGTTTAAAGACTGGGAGGAGTTGGGGGAGGAGATTAGGTTAAAGGTCTGTGAATGAAACTGATACAGCAATATTTGAAATTCCTGAGGCAAAATTTCTTCATATGGAAATTGGAGACAATGATCATATCTACCTTAGCAGATTATATAATGAATTATTTTCCTAGGGCTCCTGTAATAAAGTACCACAAACTGGGTAACTTAAGCAACAGAGATTTATTGCCTCACAATTCTAGAGGTGAGAAGTCCAGATCAAGAAGCTGGCAGGGCTAGGCTGCCTGAAAAGGTGCTAAGGAAGGAACTGTTCCAGTCCTCTTTCTCTCCTTCCAGTAGTTCCTTGGCTTGTGACAGCACAGTGTCAATTCTCATATGGCATCCTCCCCGTATGCCTCTCTCTATGTCCAAATCTCTTACTAGCAGCTTCAGTGTCTTGCAGTTGGTCTCAGCAGCCCTGGGATTCCTGTGATAGCAGCAACCATGGCTGCTAGGCTGTGCTGCCAACTGGATCCTGCGCGGGACCGTCCTTTA*
13	1802	6	*CCAAATATTGCCCAAGGTCTTACATAAGAGGACTCTTGGACTCTCAGCAATGTCAACGACCGACCTTGAGGCATACTTCAAAGACTGTTTGTTTAAAGACTGGGAGGAGTTGGGGGAGGAGATTAGGTTAAAGGTCTTTGTACTAGGAGGCTGTAGGCATAAATTGGTCTAGTGTTGCTATACATTTTTATTTTACATATGTTATAAACTCTATAATTCTGCACAGTTATTTTTTGCTTTAATCAATTATGTTTAAATAAATTAGGAAATGAGAAAATAGTCTATAATTGCTCACTTGTGATACTCTTAATTCTTTCATGTACATTCAAGTTTCTATCGGTATCATTTCTCTTCAGCTTAAAAAAACTTTTAATTTTTATTTATTTCTCATATTTTTTTTTTTTTAATACTTTAAGTTCTGGGATACATGGGCAAAACGGGCAGGTTTGTTACATAGGTATACACAGGCCAGGGCTGCTAGGCTGGGCTGCCAACTGGATCCTG*

14 ***RED = HBV sequence, BLACK = Human sequence, PURPLE = *NcoI* restriction site, BLUE = Shared sequence, ORANGE = Unknown sequence**
