# Supplementary material for: Novel digital droplet inverse PCR assay shows that natural clearance of hepatitis B infection is associated with fewer viral integrations
Source: Emerg Microbes Infect. 2025 Jan 3;14(1):2450025. doi: 10.1080/22221751.2025.2450025 (PMC11731057; doi:10.1080/22221751.2025.2450025)
Supplement: Table S2.pdf [file TEMI_A_2450025_SM0040.pdf]

Supplemental Table 2. The Sequence of Human-HBV Junction used as controls and establishment of ddinvqPCR

Clone	Downstream HBV junction	Upstream HBV junction	Chromosome	Downstream junction (BLUE = HUMAN, BLACK = HBV)	Upstream junction (BLUE = HUMAN, BLACK = HBV)
1	1821	1821	17	*GGCATACTTCAAAGACTGTTTGTTTAAAGACTGGGAGGAGTTGGGGGAGGAGATTAGGTTAAAGGTCTTTGTACTAGGAGGCTGTAGGCATAAATTGGTCTGCGCACCAGCACCATGCAATATATTGTAAATATTTTTGCTTGTTTGTAATCTTGTCTTTTAACTTTATGTTGACTTTTACCATTCAGATGCAAAAAAATTGAAGTAGTTAAATTTATCAGTGTTTTTCCTTTATGACT*	*TGAAATACTTGTGTGTTTTGGTTTTTGTTTTACTGATAGGTAAAGCATTATTTATTAAGATTTAATAAATTATTTATATTATTAATTTCCTACAGCCTCCTAGTACAAAGACTTTTTCACCTCTGCCTAATCATCTCTTGTTCATGTCCTACTGTTCAAGCCTCCAAGCTGTGCCTTGGGTGGCTTTGGGGCAT*
2	1822	1828	6	*ACTTCAAAGACTGTTTGTTTAAAGACTGGGAGGAGTTGGGGGAGGAGATTAGGTTAAAGGTCTTTGTACTAGGAGGCTGTAGGCATAAATTGGTCTGCGCACCAGCACCATGCAACGTTCAGGAGTCTATCCTTGACCTCCACCTATTCTTATTCACTATGTTCTCTTTAGCGTTTAATTGGAAGCTACAGTTTCAACTCCCACCTGTTACCAGTGACTTCCAAACCTCC*	*CTGGAGTGCAATGGTGCAATCATGGTTCATTGCAGCCTTGAACTCTTGGCCTGAAGCAATCCTCCCTCCTCAGGCTCCTGAGTCCTCTGGGATTATAGGCACACACCACCACCTCTGCCTAATCATCTCTTGTTCATGTCCTACTGTTCAAGCCTCCAAGCTGTGCCTTGGGTGGCTTTGGGGCATGGACATCGACCCTTATAAAGAATTTGGAGCTACTGTGGAGTTAC*
