# Supplementary figures and images for: Novel digital droplet inverse PCR assay shows that natural clearance of hepatitis B infection is associated with fewer viral integrations
Source: Emerg Microbes Infect. 2025 Jan 3;14(1):2450025. doi: 10.1080/22221751.2025.2450025 (PMC11731057; doi:10.1080/22221751.2025.2450025)

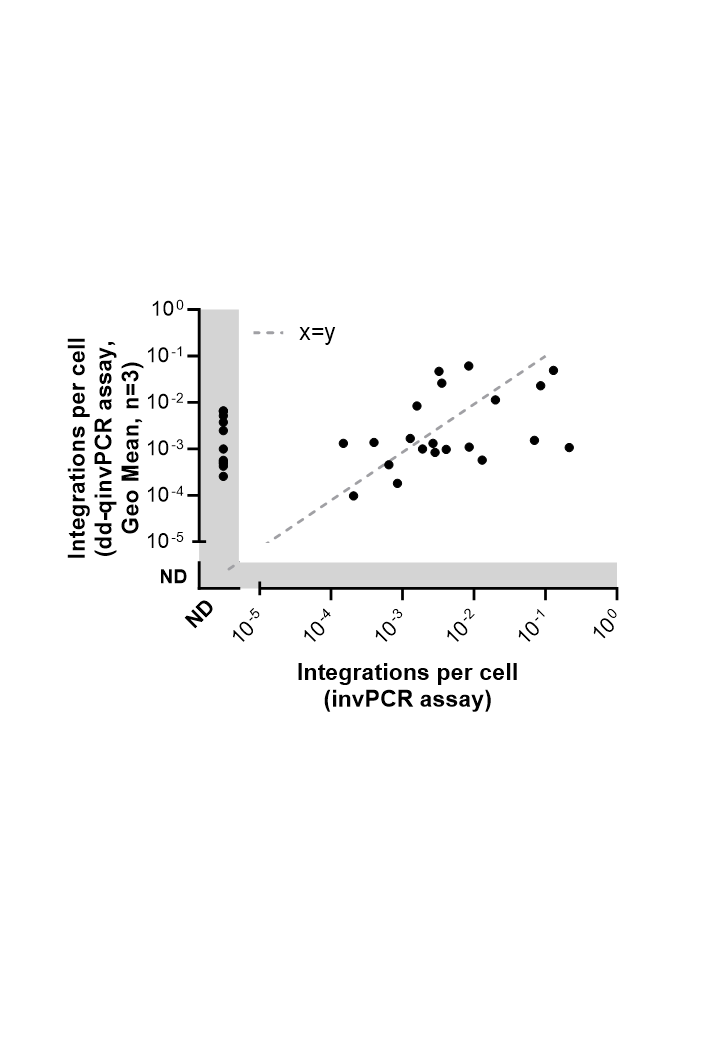

Supplement: Figure_S1.TIF [file TEMI_A_2450025_SM0039.tif]
